# Diversity Patterns of Spontaneous Plants and Their Multi-Scale Driving Mechanisms in Cold Regions: A Case of 14 Cities in Heilongjiang Province, China

**DOI:** 10.3390/plants14203145

**Published:** 2025-10-12

**Authors:** Feinuo Li, Congcong Zhao, Haiyan Zhu, Xueting Yang, Yuandong Hu

**Affiliations:** 1 College of Landscape Architecture, Northeast Forestry University, Harbin 150040, China; lifeinuo@nefu.edu.cn (F.L.); zhao_congcong@nefu.edu.cn (C.Z.); 13142619253@163.com (X.Y.); 2 College of Architecture, Tianjin University, Tianjin 300072, China; zhuhaiyan@nefu.edu.cn; 3 XAUAT-UWA International Joint Lab on Urban Biodiversity and Design, Xi’an University of Architecture and Technology, Xi’an 710055, China

**Keywords:** cold regions, spontaneous plants, diversity distribution patterns, driving mechanisms, climatic subzones, habitat types

## Abstract

Cold-climate cities remain poorly studied, yet their spontaneous flora is strongly shaped by severe winters and short growing seasons. Heilongjiang Province, the northernmost region of China, provides a valuable case study given its rapid urbanization. As an important component of urban biodiversity, the diversity distribution patterns of spontaneous plants and their underlying causes remain underexplored from multi-scale and multi-dimensional perspectives. Therefore, this study aimed to test how climatic subzones and habitat types jointly influence spontaneous plant diversity across urban landscapes in 14 cities of Heilongjiang Province. Based on vegetation surveys, we applied calculations of α- and β-diversity, along with hierarchical clustering, across climatic subzones, cities, and habitat types to elucidate the diversity patterns and their multi-scale driving mechanisms. The results showed the following: (1) A total of 778 spontaneous plant species were recorded, belonging to 98 families and 395 genera. Native plants accounted for 58.7%, and non-native plants accounted for 41.3% (including 77 invasive species). (2) Perennial herbs dominated overall (45.2%), consistent with winter filtering, whereas annual/biennial herbs were more common in warmer subzones such as II B2. (3) Forest gaps (FG) and shrub–grassland gaps (SG) supported the most diverse spontaneous plant communities, highlighting habitat heterogeneity. (4) Species richness peaked in subzone II B2 and was lowest in subzone I A1, while abandoned land (SA) and shrub–grassland gaps (SG) supported the richest communities. (5) β-diversity analyses indicated homogenization under extreme cold in subzone I A1 and greater turnover in warmer subzone II B2, reflecting contrasting climatic filters. The “light patches” in FG habitats and the “disturbance filtering” in LA habitats further shaped the differences in local communities. This study reveals the diversity distribution patterns and adaptation strategies of spontaneous plants in cold cities, emphasizing their integration into urban planning while addressing the dominance of invasive species.

## 1. Introduction

Habitat fragmentation triggered by rapid urbanization is accelerating biodiversity loss worldwide, particularly in high-latitude regions with fragile ecosystems, where this process is especially pronounced [1,2]. Cold region ecosystems, subjected to long-term low-temperature stress and compounded human activities, face severe challenges in maintaining biodiversity and ecological stability, primarily from extreme climatic constraints, habitat fragmentation due to urbanization, and increased pressure from biological invasions. Spontaneous plants, defined as urban vegetation that neither belongs to remnants of natural vegetation nor is deliberately cultivated by humans, have attracted increasing attention in this context. Against this backdrop, spontaneous plants—which typically require no artificial introduction or maintenance and can grow spontaneously in heterogeneous urban habitats such as wall cracks, road edges, and gaps in hard pavements—have emerged as a key component in enhancing the resilience of urban ecosystems [3,4,5,6,7]. These plants, due to their strong environmental adaptability and reproductive capacity, form low-maintenance plant communities with multiple critical ecological functions, including carbon sequestration, oxygen release, mitigation of the urban heat island effect, and maintenance of urban pollination networks [8].

Research on urban spontaneous plants has evolved from species cataloging to the analysis of maintenance mechanisms on a global scale [5,9,10,11,12,13,14]. Existing studies have found that urban environments drive phenotypic adaptations in plants (e.g., leaf morphology), and that factors like habitat fragmentation and disturbance frequency regulate community structure, as evidenced by reports that tropical communities are influenced by human disturbance, temperate plant richness correlates with precipitation, and urban transportation enhances species dispersal efficiency [3,7,15,16]. Additionally, related studies have shown that plants in wastelands improve soil nutrient availability through root activity, and naturally successional communities exhibit higher ecosystem multifunctionality compared to artificially planted vegetation [14,17]. Conceptual and review studies have emphasized the conservation and functional significance of spontaneous urban vegetation [5,10], while empirical work from northern Europe shows that spontaneous species can dominate engineered or abandoned substrates (e.g., green roofs, wastelands), with climatic factors such as freeze–thaw cycles and mean annual temperature shaping persistence and composition [12,13]. Surveys across Nordic cities further document distinct patterns in urban tree diversity and management, highlighting differences between street and park populations and varying proportions of native versus non-native taxa, and underline the need to consider paved-site stress tolerances in tree selection [9,11]. These findings provide a solid foundation for understanding the distribution patterns and driving mechanisms of spontaneous plants across different climatic zones and habitat types. However, domestic research in this field started relatively late and is highly uneven in spatial distribution, primarily focusing on large and medium-sized cities in warm temperate and subtropical zones (such as Beijing, Shanghai, Kunming) [18,19,20,21,22,23,24,25]. Taking Heilongjiang Province, the highest-latitude and most climatically severe region in China, as an example, existing research mainly concentrates on individual cities like Harbin, with no systematic understanding of the species composition and diversity patterns of urban spontaneous plants at the provincial scale [26,27]. These limitations in study number and spatial scale have constrained our understanding of plant community assembly and maintenance processes in cold urban ecosystems. Moreover, cold-climate urban ecosystems are likely to differ mechanistically from warmer regions due to the unique effects of winter filtering and short growing seasons, highlighting the scientific novelty of studying spontaneous plants in cold regions. These limitations in research scale and spatial distribution severely constrain the understanding of plant community assembly mechanisms and maintenance processes in cold urban ecosystems.

This study targets Heilongjiang Province, a region classified as a typical cold temperate zone according to the Chinese National Standard GB/T 17297-2021 (Climate Regionalization of China) [28], the highest-latitude region in China with distinct climatic gradients, and systematically investigates 14 cities across the province, achieving comprehensive coverage of the five climatic subzones and major urban types at the provincial scale. Based on cataloging the species composition, community types, and life-form structure of urban spontaneous plants, this study further analyzes the distribution patterns of their α-diversity and β-diversity at urban, climatic subzone, and habitat scales. We hypothesize that the assembly of spontaneous plant communities in cold-temperate cities is predominantly driven by a hierarchy of filters, where broad-scale climatic conditions constrain the species pool, and local habitat heterogeneity fine-tunes community composition. To test this overarching hypothesis, this research aims to address the following: (1) the diversity composition and spatial distribution patterns of urban spontaneous plants in Heilongjiang Province; (2) the driving mechanisms of climatic subzones and local habitat scales on the distribution patterns of urban spontaneous plants; and (3) diversity maintenance and management strategies for urban spontaneous plants. The findings are expected to provide critical data and a scientific basis for the conservation and management of biodiversity in cold regions.

## 2. Results

### 2.1. Spontaneous Plant Composition

**Figure 1:**
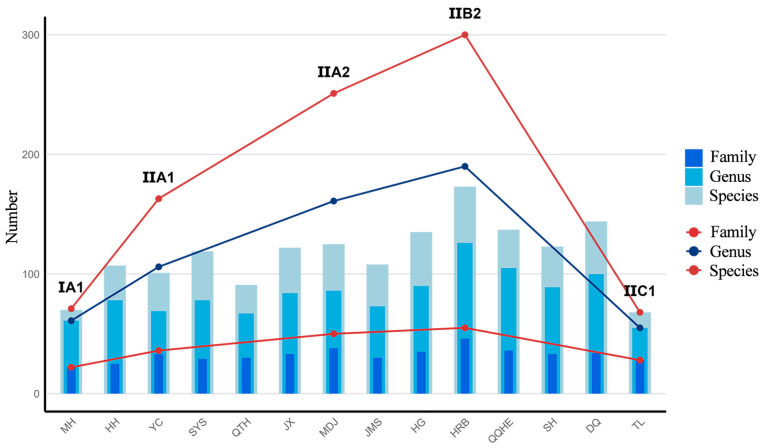
Species Composition of 14 cities and 5 subzones. Note: For the full names of the city and climatic subzone abbreviations, refer to Figure 15 in the Methodology Section 4.1.

**Figure 2:**
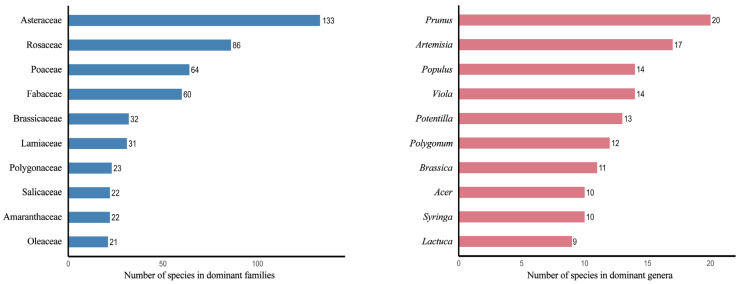
Top 10 families and genera by species number.

A total of 778 spontaneous plant species belonging to 98 families and 395 genera were recorded across the 14 cities in Heilongjiang Province, with significant differences in species diversity composition among climatic subzones (Figure 1). The I A1 subzone exhibited relatively low plant species richness, with Mohe recording 70 species belonging to 22 families and 61 genera, reflecting the limiting effects of extreme cold on plant distribution. Species diversity significantly increased in the II A2 subzone, where Mudanjiang recorded 251 species belonging to 50 families and 161 genera, likely due to its more complex ecological environment. The II B2 subzone showed the highest species richness, with Harbin reaching 300 species belonging to 55 families and 190 genera, possibly attributable to its more diverse and complex ecosystem structure. At the provincial level, Asteraceae, Rosaceae, Poaceae, and Fabaceae were the dominant families, while genera such as *Prunus*, *Artemisia*, and *Populus* were the dominant genera, collectively reflecting both commonalities and differences in the taxonomic characteristics of plants in this region (Figure 2).

**Figure 3:**
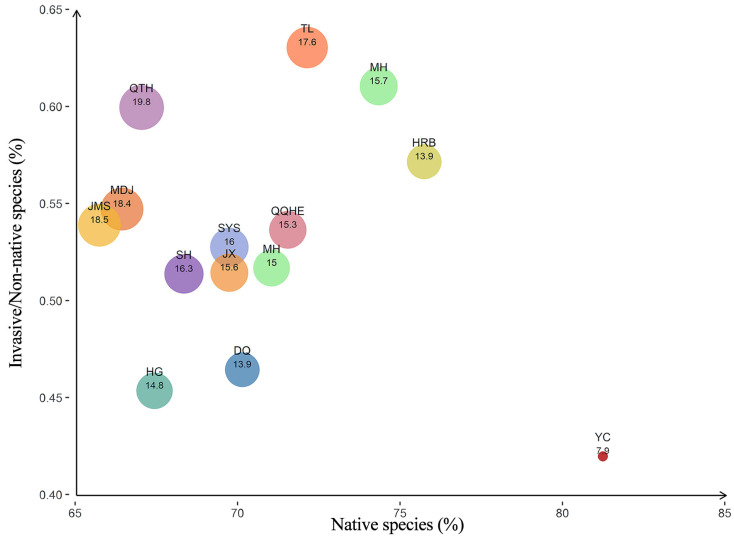
Species origin of spontaneous plants in 14 cities. Note: 1. Bubble size means invasive plants/Total species (%). 2. Species origin (native, non-native, invasive) was determined through comprehensive consultation of authoritative sources, including the Flora of China, official catalogs of native species from the National Forestry and Grassland Administration, and specialized studies on invasive flora (e.g., The Inventory of Invasive Alien Plants in China, 2023).

A total of 490 native spontaneous plant species (58.7%) and 288 non-native spontaneous plant species (41.3%) were recorded across the 14 cities in Heilongjiang Province (Figure 3). Among the non-native plants, 77 species were identified as invasive, accounting for 9.9% of the total species and 26.7% of the non-native plants. The proportion of native spontaneous plants was generally high in all cities, with Yichun having the highest proportion at 81.2%. The proportions of invasive species among non-native species were relatively elevated in Tailai, Mohe, and Qitaihe, reaching 63.2%, 61.1%, and 60.0%, respectively. As the provincial capital, Harbin exhibited a high proportion of native spontaneous plants (approximately 75.7%), but invasive species accounted for 57.1% of the non-native spontaneous plants, indicating strong pressure from alien species invasion in its ecosystem. Overall, native plants dominate, but the varying distribution of invasive species reflects differences in the ecological health of the cities.

**Figure 4:**
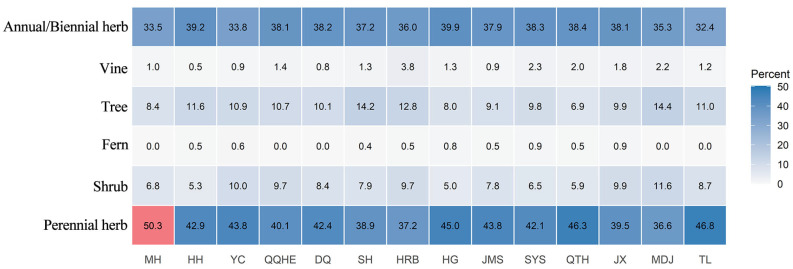
Life form composition of spontaneous plants in 14 cities.

Perennial herbs were the dominant life form among spontaneous plants (Figure 4, accounting for 45.2% of the total species, followed by annual/biennial herbs (31.0%)). Across all cities, the proportion of perennial herbs ranged between 37.2% and 50.3%, while annual/biennial herbs accounted for 32.4% to 39.9%. Trees constituted 6.9% to 14.4%, and shrubs represented 6.8% to 11.6%. Vines (0.5–3.8%) and ferns (0–0.9%) both exhibited relatively low proportions. Among 14 cities, Mohe had the highest proportion of perennial herbs, which can be attributed to its distinct and exceptionally cold climatic conditions.

**Figure 5:**
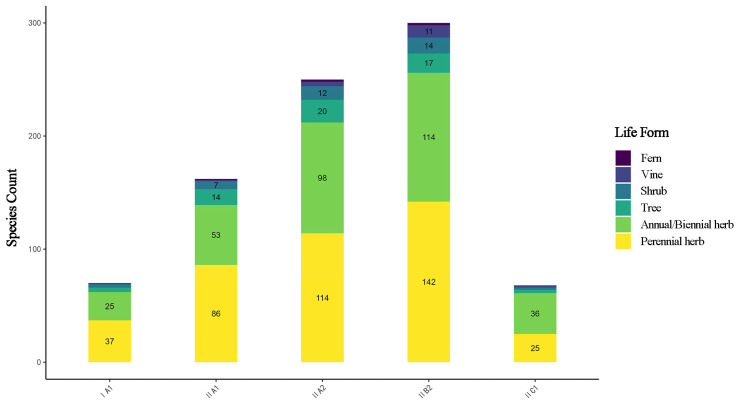
Life form composition of spontaneous plants in 5 subzones.

The life form composition of spontaneous plants exhibited distinct variations across different climatic subzones (Figure 5). Perennial herbs were most abundant in subzone IIB2 (142 species), while annual herbs were similarly rich in both subzones II B2 and II A2. Trees and shrubs showed relatively even distribution across subzones. Vines and ferns were generally scarce throughout the province. Overall, herbaceous plants dominated in all climatic subzones, and the life form structure exhibited significant variations corresponding to climatic conditions.

### 2.2. Classification of Community Types and Their Distribution Characteristics

#### Clustering Results and Community Type Classification

**Figure 6:**
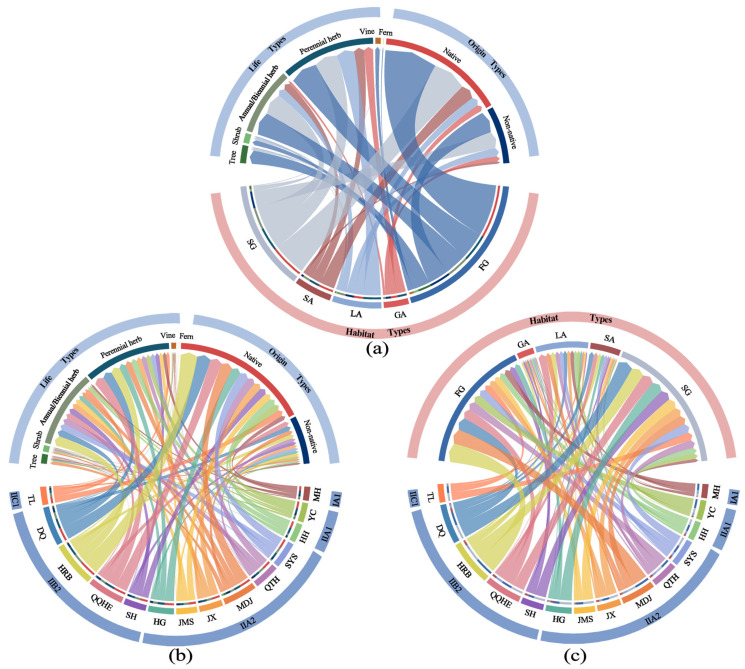
(**a**) Relationships among life forms and origin types, habitat types, and dominant spontaneous plant community types; (**b**) Relationships among life forms and origin types, corresponding climatic subzones, and dominant spontaneous plant community types across different cities; (**c**) Relationships among habitat types, corresponding climatic subzones, and dominant spontaneous plant community types across different cities. Note: Each colored arc (node) represents a category (e.g., a habitat type, a city), and its length is proportional to the number of dominant spontaneous plant community types within that category. The connecting ribbons (chords) between nodes represent associations between categories, with the ribbon width proportional to the strength of the association.

The Euclidean distance matrix of community relative dominance clustering showed significant phenetic correlation with the original data (Mantel r = 0.8842, *p* < 0.001), and the optimal number of clusters was 218 (Figure S1). Three chord diagrams drawn on this basis reveal the characteristics of community composition and spatial distribution patterns across different ecological dimensions (Figure 6). Figure 6a shows that forest gap (FG, 88 groups) and shrub-grassland gap (SG, 61 groups) habitats exhibited the richest community types, though ferns were only observed in FG habitats, while gravel-type abandoned land (GA) and soil-type abandoned land (SA) were dominated by herbaceous plants. Across all habitat types, the number of native plant groups was generally higher than that of non-native groups, with all non-native plants in GA habitats being invasive species. Differences in spontaneous plant community structures among cities are illustrated in Figure 6b. Cities such as Harbin, Daqing, and Qiqihar had the highest number of community groups, with perennial herb communities dominating. Tree community types were predominantly concentrated in cities within subzones II A2 and II B2. Native plant groups dominated across all cities. As shown in Figure 6c, FG and SG habitat types were widely distributed in all cities, representing typical urban habitat types. Overall, cities such as Harbin, Mudanjiang, and Shuangyashan exhibited the highest diversity of spontaneous plant community types and habitat type combinations, reflecting greater community heterogeneity and habitat complexity.

### 2.3. Distribution Patterns of α and β Diversity Indices of Spontaneous Plants Across Multiple Scales and Dimensions

#### 2.3.1. α-Diversity Index

##### Influence of Climate on the Distribution of α-Diversity Index

**Figure 7:**
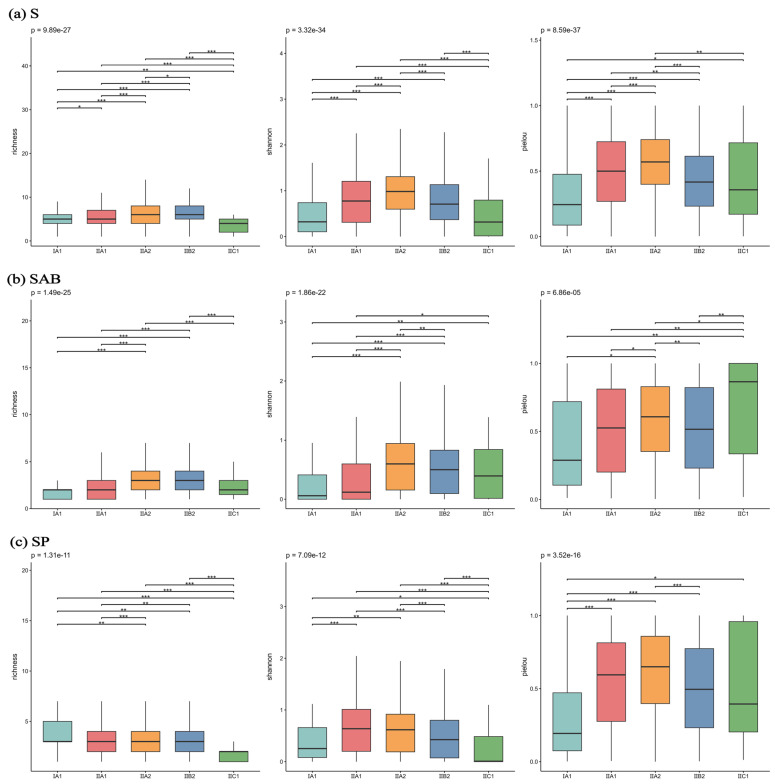
α-diversity index of different spontaneous plant types across climatic subzones. Note: S-All spontaneous plants; SAB-Spontaneous annual and biennial plants; SP-Spontaneous perennial plants. *** *p* < 0.001; ** *p* < 0.01; * *p* < 0.05.

The distribution characteristics of α-diversity indices of spontaneous plants varied significantly across different climatic subzones (Figure 7). The spatial patterns of species richness and Shannon index for all spontaneous plants and for annual/biennial plants were similar: subzone II B2 exhibited the highest richness index, while subzone I A1 showed the lowest, with extremely significant differences between I A1 and II A2/II B2 (*p* < 1 × 10^−6^). The Shannon index was highest in subzone II A2, significantly higher than in I A1 (*p* < 0.001). For annual/biennial plants, the Pielou evenness index was highest in subzone II C1, significantly superior to that in I A1. In contrast, perennial plants showed the opposite trend: I A1 had the highest richness, significantly higher than I IC1 (*p* < 1 × 10^−10^); II A1 exhibited the highest Shannon index, while II C1 had the lowest; II A2 showed the highest Pielou index, while I A1 had the lowest.

##### Influence of Habitat Types on the Distribution of α-Diversity Index

**Figure 8:**
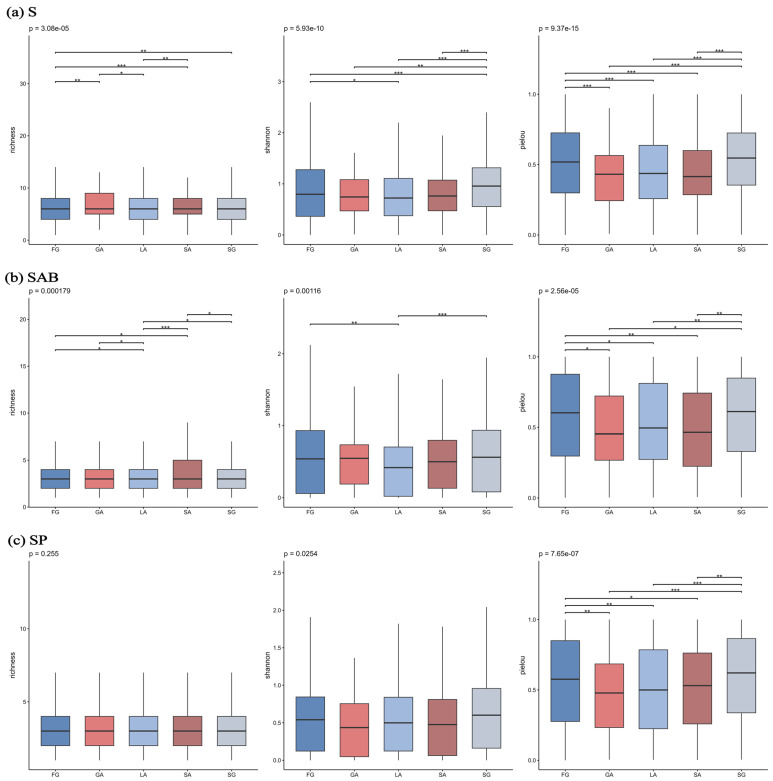
α-diversity index of different plant types across habitat types. Note: *** *p* < 0.001; ** *p* < 0.01; * *p* < 0.05.

Habitat types significantly affected the α-diversity of spontaneous plants (*p* < 0.05) (Figure 8). All spontaneous plants and annual/biennial plants exhibited similar distribution patterns in richness and Shannon indices: soil-type abandoned land (SA) habitats showed the highest richness index, significantly higher than lawn (LA) habitats; shrub-grassland gap (SG) habitats exhibited the highest Shannon index; and both groups had higher evenness indices in SG and forest gap (FG) habitats. For perennial spontaneous plants, the differences in richness and Shannon indices across habitats were not significant, but their evenness index was highest in SG habitats and superior to all other habitat types.

#### 2.3.2. β-Diversity Index

##### Differences in Community β-Diversity Index Across Climatic Subzones

(1) S-Type Communities

**Figure 9:**
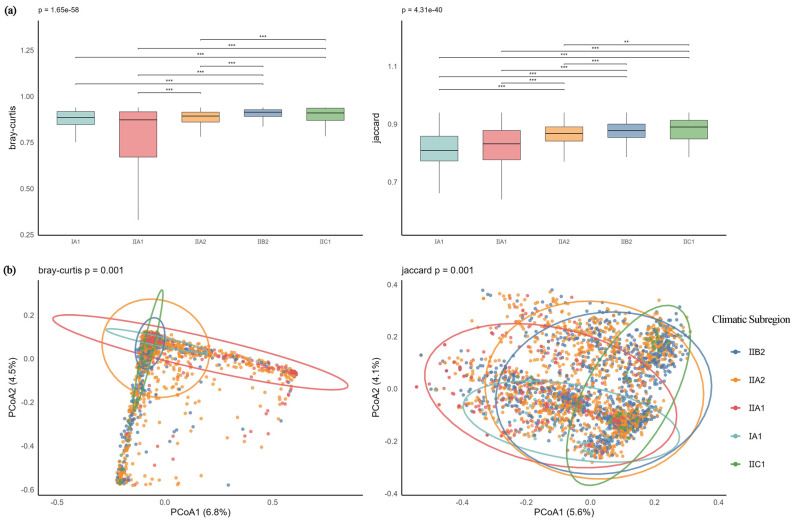
(**a**) Boxplots of Bray–Curtis and Jaccard distances for S-type communities across climatic subzones; (**b**) PCoA analysis of S-type communities across climatic subzones. Note: *** *p* < 0.001; ** *p* < 0.01.

Boxplot results of within-group distances based on Bray–Curtis and Jaccard distances (Figure 9a) showed that under Jaccard distance, the I A1 group had the lowest median within-group distance (median = 0.8689), indicating relatively stable and similar species composition among samples within this subzone. Under Bray–Curtis distance, the II A1 group exhibited the lowest median within-group distance (median = 0.9329), suggesting consistent community abundance structure. In contrast, the II B2 and II C1 groups generally had higher median within-group distances under both distance metrics, indicating greater heterogeneity and structural variability among samples within these communities. PCoA results (Figure 9b) revealed that the first two axes explained 11.3% and 9.7% of the variance under Bray–Curtis and Jaccard distances, respectively. This level of explanatory power is consistent with expectations for heterogeneous ecological data. Sample points from different groups showed some separation, reflecting the distinguishing effect of climatic subzones on community structure, which was further supported by PERMANOVA analysis (Bray–Curtis: F = 14.02, *p* = 0.001; Jaccard: F = 16.20, *p* = 0.001). In summary, for S-type communities, climatic subzones significantly influenced both species composition and abundance structure, manifesting as systematic differences in community stability and structural variability across subzones. This highlights the critical role of climatic zonation in shaping regional community β-diversity.

(2) SAB-Type Communities

**Figure 10:**
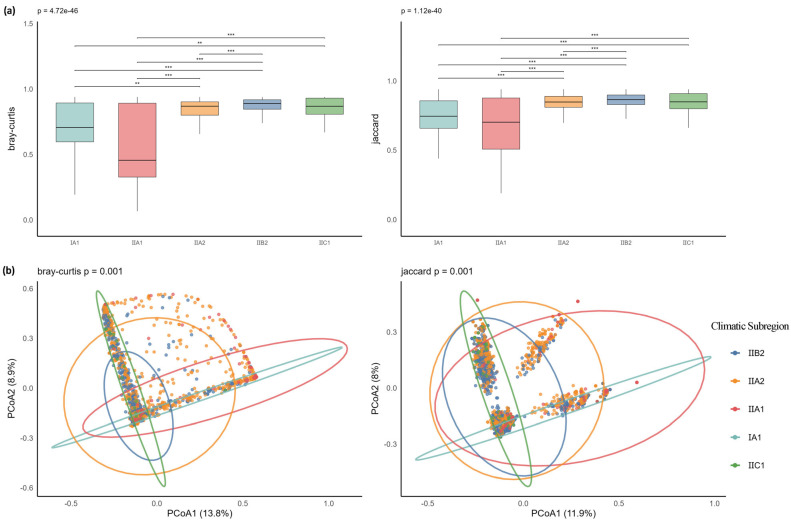
(**a**) Boxplots of Bray–Curtis and Jaccard distances for SAB-type communities across climatic subzones; (**b**) PCoA analysis of SAB-type communities across climatic subzones. Note: *** *p* < 0.001; ** *p* < 0.01.

Based on calculations using both distance metrics (Figure 10a), the II A1 group exhibited the lowest median distances under both Bray–Curtis (0.516) and Jaccard (0.763) measures, indicating relatively consistent community structure among samples within this group in terms of both species composition and abundance distribution, and demonstrating high stability. In contrast, the II B2 group showed the highest median distances under both metrics (Bray–Curtis: 0.950; Jaccard: 0.925), with significant differences compared to other groups (*p* < 0.001), suggesting greater variability in community structure and notable differences among samples. PCoA visualization results (Figure 10b) revealed clear clustering trends of sample points from different climatic subzones, with good separation between groups. The first two axes explained 22.7% and 19.9% of the variance under Bray–Curtis and Jaccard distances, respectively. This result was further validated by PERMANOVA analysis (Bray–Curtis: F = 19.31, *p* = 0.001; Jaccard: F = 18.13, *p* = 0.001).

(3) SP-Type Communities

**Figure 11:**
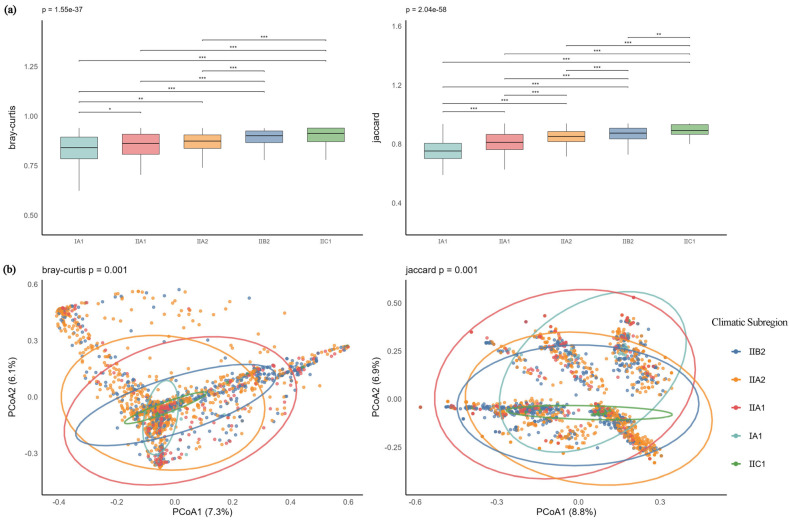
(**a**) Boxplots of Bray–Curtis and Jaccard distances for SP-type communities across climatic subzones; (**b**) PCoA analysis of SP-type communities across climatic subzones. Note: *** *p* < 0.001; ** *p* < 0.01; * *p* < 0.05.

Based on within-group distance calculations using both metrics (Figure 11a), the I A1 group exhibited the lowest median distances under both Bray–Curtis (0.901) and Jaccard (0.813) measures, indicating the most consistent community structure among samples within this group, with significant differences compared to the other four groups (*p* < 0.05). The II C1 group showed the highest median distances (Bray–Curtis: 0.973; Jaccard: 0.952), with maximum values reaching 1, suggesting the greatest internal variability and significantly higher variation in community composition and abundance distribution compared to other groups. PCoA results (Figure 11b) revealed that the first two axes explained 13.4% and 15.7% of the variance under Bray–Curtis and Jaccard distances, respectively. PERMANOVA results aligned with the visualization: I A1 showed significant differences from all other groups, consistent with the boxplot findings. However, the difference between II C1 and II B2 was not significant under Bray–Curtis (*p* = 0.09) but significant under Jaccard (*p* = 0.008), indicating similarity in abundance patterns but divergence in species composition between these two groups.

##### Analysis of β-Diversity Differences Across Habitat Types

(1) S-Type Communities

**Figure 12:**
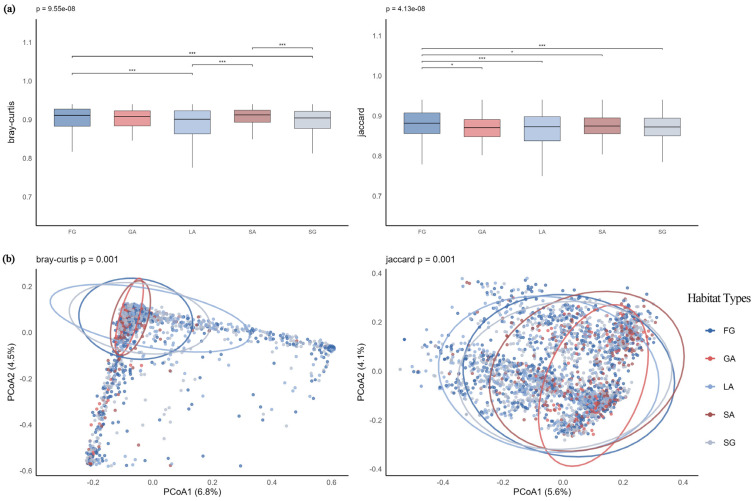
(**a**) S-type communities Bray–Curtis and Jaccard distances across habitats; (**b**) PCoA of S-type communities across habitats. Note: *** *p* < 0.001; * *p* < 0.05.

Boxplot analysis results (Figure 12a) showed that forest gap (FG) habitats exhibited the highest median within-group distance and the widest variability range, indicating significant differences in community composition. Dunn’s test results further revealed that under Jaccard distance, FG habitats differed significantly from all other habitat types. PCoA analysis (Figure 12b) demonstrated that the first two axes explained 11.3% and 9.7% of the variance under Bray–Curtis and Jaccard distances, respectively. Although the explanatory power was limited, PERMANOVA results confirmed significant differences in community structure among habitat types (Bray–Curtis: F = 5.64, *p* = 0.001; Jaccard: F = 6.85, *p* = 0.001). Gravel-type abandoned land (GA) and soil-type abandoned land (SA) habitats exhibited stable and consistent community composition, while FG habitats showed significant variability and pronounced differences in community structure.

(2) SAB-Type Communities

**Figure 13:**
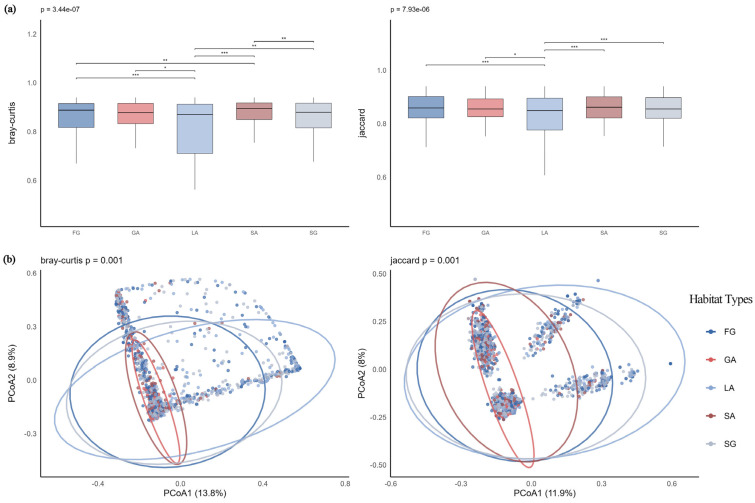
(**a**) SAB-type communities Bray–Curtis and Jaccard distances across habitats; (**b**) PCoA of SAB-type communities across habitats. Note: *** *p* < 0.001; ** *p* < 0.01; * *p* < 0.05.

Analysis of within-group distances based on both distance metrics (Figure 13a) revealed that lawn (LA) habitats exhibited the lowest median within-group distances under both measures, indicating relatively discrete composition and significant to highly significant differences compared to the other four habitat types. In contrast, soil-type abandoned land (SA) habitats showed the highest median within-group distances under both metrics, suggesting greater structural stability and higher internal similarity. These conclusions were further supported by PCoA visualization (Figure 13b). PERMANOVA results confirmed that habitat type significantly influenced community composition (Bray–Curtis: F = 7.76, *p* = 0.001; Jaccard: F = 7.63, *p* = 0.001). The first two axes explained 22.7% and 19.9% of the variance under Bray–Curtis distance, and 11.9% and 8.0% under Jaccard distance, respectively. Community variations in gravel-type abandoned land (GA) and SA habitats were primarily concentrated along specific directional gradients, with relatively stable compositional structures.

(3) SP-Type Communities

**Figure 14:**
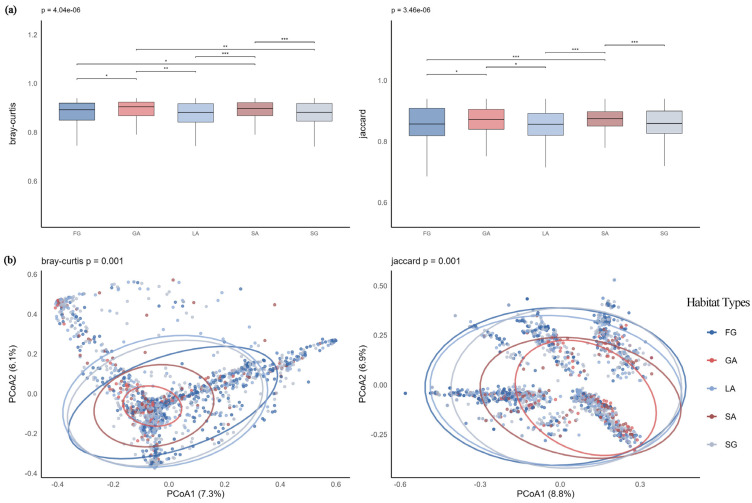
(**a**) SP-type communities Bray–Curtis and Jaccard distances across habitats; (**b**) PCoA of SP-type communities across habitats. Note: *** *p* < 0.001; ** *p* < 0.01; * *p* < 0.05.

Boxplots of within-group distances under both metrics (Figure 14a) revealed that gravel-type abandoned land (GA) and soil-type abandoned land (SA) habitats exhibited the highest median within-group distances, indicating substantial community divergence and high structural dispersion among samples. Under Bray–Curtis distance, both habitats showed significant differences (*p* < 0.05) compared to the other three habitat types. PCoA results (Figure 14b) demonstrated that the first two axes collectively explained 13.4% and 15.7% of the community structure variance under Bray–Curtis and Jaccard distances, respectively. PERMANOVA analysis confirmed that habitat type significantly influenced the community structure of perennial spontaneous plants (Bray–Curtis: R^2^ = 0.0098, F = 6.11, *p* = 0.001; Jaccard: R^2^ = 0.0107, F = 6.66, *p* = 0.001). Sample points from GA habitats showed relatively clustered distribution in the two-dimensional ordination space, indicating consistent community structure, while FG habitat samples were widely distributed, reflecting greater compositional variability.

## 3. Discussion

### 3.1. Characteristics of Species Composition and Potential Adaptation-Related Interpretations of Spontaneous Plants in Heilongjiang Province

The species composition of spontaneous plants in Heilongjiang Province (778 species, with native plants accounting for 58.7% and perennial herbs dominating) reflects both the common characteristics of temperate cities and the unique filtering effects of cold environments. In terms of family and genus characteristics, the dominance of Asteraceae, Rosaceae, Poaceae, and Fabaceae aligns not only with findings from Beijing and Zhengzhou [29,30] but also with studies in other globally representative urban areas such as Shanghai, Yunnan, and Kyrgyzstan [24,25,31], indicating the broad adaptability of cosmopolitan families to specialized urban habitats. Compared to most warm temperate cities, cold-region urban areas further rely on cold-resistant phenotypic traits of Asteraceae plants to cope with low temperatures, forming more unique adaptation strategies [32,33]. Genera such as *Artemisia* and *Prunus*, as dominant genera in Heilongjiang Province, exhibit even more pronounced adaptive features. For instance, the leaf trichomes of *Artemisia* reduce water loss under low temperatures and strong winds, while the thickened cuticles of *Prunus* enhance frost resistance in dry and cold climates. These phenotypic adjustments are results of adaptive evolution under long-term low-temperature stress [34].

The species richness gradient across climatic subzones in Heilongjiang Province (300 species in subzone II B2 vs. 70 species in subzone I A1) may be related to “water-thermal synergy”: the longer growing season and higher precipitation in the mid-temperate Songliao region (II B2) likely provide a more stable resource base for species, while the extreme cold in the north temperate Genhe region (I A1) may restrict the distribution of most species through “low-temperature filtering”, allowing only cold-tolerant native species to persist. This gradient difference is significantly more pronounced than in other warm temperate cities, highlighting the strong constraining effect of cold climates on species pools. This pattern aligns with findings from the dry-hot valley of the Yuanjiang River [35]. We propose that a key difference in cold regions is the potentially more prominent role of the urban heat island effect in extending the growing season [36], suggesting a plausible mechanism for the observed invasion success [37]. Compared to Yunnan Province, a biodiversity hotspot where native plants account for 73.4% [25]. Heilongjiang has a slightly lower proportion of native species. However, its cold-tolerant native species exhibit stronger stress resistance, holding unique significance for agricultural production and ecological restoration.

The spatial differentiation of life forms in spontaneous plants further reveals their adaptation strategies: Mohe (I A1) exhibits the highest proportion of perennial herbs, likely relying on underground rhizomes for nutrient storage and increased soluble sugars and other organic compounds to overwinter—a “stress avoidance strategy” to evade extreme cold seasons [38,39]. This life form structure, dominated by perennial herbs (45.2%), represents a core characteristic of cold-region flora and aligns with the life form features of threatened plants in Kyrgyzstan [31]. The overwintering strategy of underground organs is consistent with the “freezing resistance regulation mechanism” of cold-region plants [40]. In contrast, Harbin (II B2) shows richness in annual and biennial herbs, potentially employing a “fast-growing strategy” to capitalize on resource pulses during the growing season [32,36,41,42]. The short growing season in cold regions limits the growth cycle of vine plants.

The high invasion ratio in cold-region habitats may be related to disturbance intensity. As the provincial capital and a major transportation hub, frequent human-mediated introductions in Harbin are likely responsible for its high proportion of invasive species. The high proportion of invasive species among the non-native flora in Tailai and Mohe (>60%) may reflect the presence of underutilized resources (“empty ecological niches”) in the exposed habitats of wastelands (GA/SA), which are readily exploited by invasive species adapted to low-resource environments [43,44]. This aligns with findings from Beijing, where “low-maintenance habitats were associated with higher invasion risks” [29].

### 3.2. Multi-Scale Driving Mechanisms of Spontaneous Diversity Distribution Patterns: Synergistic Effects of Climatic Gradient and Habitat Heterogeneity

#### 3.2.1. Driving Factors of α-Diversity Index

From the perspective of climatic subzones, the urban heat island effect in subzone II B2 extends the growing season, providing a longer resource window for the rapid germination and reproduction of annual/biennial plants. Coupled with the microhabitat diversity caused by habitat fragmentation, this collectively enhances species richness [35]. Meanwhile, temperature factors represented by climatic subzone divisions significantly influence α diversity across all three life forms. The high diversity of perennial plants in subzone I A1 may stem from the “niche compression under stress” theory [45].

From the perspective of habitat types, the richness of annual/biennial plants in SA habitats may result from soil disturbances (e.g., construction or abandoned farming) creating “empty ecological niches,” which provide colonization opportunities for pioneer species [46]. The high Shannon index and evenness in SG habitats may benefit from the light-moisture gradient formed by shrub and herb layers, enabling the coexistence of species with varying shade tolerance. The optimal evenness of perennial plants in SG habitats might be due to weaker influence of shrub roots on the soil surface, allowing more balanced resource allocation for perennial plants with different resource-use strategies. This pattern aligns with the intermediate disturbance hypothesis, which suppresses the overdominance of competitive species while allowing more moderately competitive species to persist [47].

Overall, the α-diversity of annual/biennial spontaneous plants is more dependent on the growing season length of climatic subzones and local high-intensity disturbances, whereas perennial plants are more driven by niche differentiation under low-temperature stress and moderately disturbed habitats. This reflects the selective response of life-form functional traits to driving factors. This conclusion, derived from diversity drivers, further corroborates the adaptive strategy of perennial herbs inferred from their life form distribution patterns discussed earlier (Section 3.1), highlighting the consistency of perennial plant responses to cold stress across different analytical approaches.

#### 3.2.2. Driving Factors of β-Diversity Index

At the climatic subzone scale, β-diversity patterns highlight the dual roles of environmental filtering and structural complexity. Subzone I A1 exhibits the lowest Jaccard distance, reflecting strong homogenization. Here, extreme cold and a shortened growing season act as powerful filters, directly constraining species survival and compressing reproductive opportunities. As a result, only a handful of broadly adaptable, cold-tolerant species persist across habitats, leading to convergent community composition [48]. In contrast, subzone II B2 shows the highest Bray–Curtis distance, where a milder climate enables pronounced vertical stratification across tree, shrub, and herb layers. This stratification, together with the naturally sparse woody layers typical of cold regions, reduces competitive exclusion and opens niche space for differentiation, particularly within the herb layer [49]. These contrasting filters also drive life-form-specific responses: annual and biennial plants, due to their r-selected strategies, display significant sensitivity to variation in growing season length, while perennial plants exhibit peak variability in the climatically volatile semi-arid subzone IIC1 [50,51].

Regarding habitat type regulation, FG habitats show significant community differences, likely resulting from canopy density variations creating “light patches,” reflecting a “multi-path assembly” mechanism where environmental filtering, dispersal limitation, and interspecific competition jointly drive community differentiation [22]. LA habitats differ significantly from other habitats in community structure, potentially due to “disturbance filtering” caused by frequent trimming. Furthermore, habitats with more complex patch shapes (SA/GA) in cold regions support higher heterogeneity due to “edge effects”, aligning with Fahrig’s finding that habitat fragmentation significantly increases (76%) habitat diversity [52].

### 3.3. Implications and Recommendations for the Maintenance and Management of Spontaneous Plant Diversity in Cold Regions

#### 3.3.1. Conservation and Management of Spontaneous Plant Diversity in Different Habitat Types

- Lawns (LA): Annual/biennial plants in this habitat exhibit significant differences compared to other habitats. It is recommended to prioritize the retention of stress-tolerant native herbaceous species and reduce trimming frequency to minimize disturbance. The “land-sharing” model of Berlin community gardens [53] can be adopted, where reducing localized impervious surfaces and promoting the coexistence of cultivated and spontaneous plants can maximize the biodiversity value of lawns.
- Shrub-Grassland Gaps (SG): This habitat generally exhibits optimal α-diversity. It is advised to maintain the existing shrub-herb structure, reduce maintenance intensity to avoid over-clearing, and utilize canopy heterogeneity to create diverse microhabitats that facilitate the dispersal of native species [54]. This approach provides direct reference value for managing SG habitats.
- Forest Gaps (FG): These habitats show the greatest variability in community composition, and ferns are exclusively found here. To reduce competition from invasive species, shade-tolerant native plants can be moderately reintroduced to enhance forest layer complexity. Additionally, protecting the litter layer and recognizing the positive role of fallen log microhabitats are recommended, especially in the I A1 subzone (Greater Khingan Range area). Fallen logs reduce surface radiative cooling, decreasing soil freezing depth by 20–30% and extending the growing season by approximately 1–2 weeks, thereby providing “microclimatic refugia” for cold-tolerant species [29].
- Soil-Type Abandoned Land (SA) and Gravel-Type Abandoned Land (GA): Both are degraded habitats resulting from human disturbance. The core restoration logic involves using native species to rapidly occupy ecological niches [23]. SA has the highest overall species richness, dominated by annual/biennial plants, and relatively intact soil substrate. It is recommended to prioritize the introduction of native pioneer species of the same life form to synergistically compete with existing vegetation and reduce invasive species’ survival space. GA, characterized by a gravel substrate, has stable communities but all non-native plants are invasive species. Restoration efforts should focus on drought tolerance and soil improvement functions, selecting cold- and drought-tolerant native species. Plant roots can enhance water and nutrient retention in the substrate, creating more stable growth conditions that feedback to promote plant prosperity, thereby forming a synergistic progression between roots and substrate.

#### 3.3.2. Regulation Strategies for Spontaneous Plant Diversity in Different Climatic Subzones

- Subzones I A1 and II A1: Both are located in the Greater and Lesser Khingan Mountains regions, with perennial plants as the core of their communities. Subzone I A1 has the highest proportion of perennial herbs but the lowest species richness, with cold tolerance being its key community characteristic. The principle of “protection priority” should be adopted to minimize human disturbance to natural patches at urban edges and maintain habitat integrity. Subzone II A1 exhibits the highest Shannon index for perennial plants and strong community stability. The focus should be on “enhancing connectivity” by preserving existing habitat heterogeneity, constructing ecological corridors to link fragmented green spaces, providing migration pathways for native species, and promoting seed dispersal to enhance community resilience [27,55].
- Subzones II A2 and II B2: Both are situated in mid-temperate zones and host larger cities in Heilongjiang Province. Subzone II A2 has high species diversity and relatively balanced life form distribution. It is recommended to delineate ecological conservation redlines, establish multi-layered vegetation structures (tree-shrub-herb) [56,57], strengthen natural maintenance capacity of communities, and prevent habitat fragmentation. Subzone IIB2 has the richest species diversity but the greatest internal community variability and faces significant invasion pressure. The strategy should focus on “connectivity and prevention,” controlling urban warming, curbing the spread of non-native species, and implementing quarterly invasive species coverage surveys [25,36,58].
- Subzone II C1: This subzone shows the highest variability in perennial plants and a high proportion of invasive species. It is recommended for plant drought-tolerant native species to improve soil quality, reduce exposed habitats, and enhance community resistance. Additionally, vertical habitats such as building gaps and walls should be utilized to retain drought- and cold-tolerant spontaneous plants, creating green spaces in vertical dimensions [59]. The “low-maintenance spontaneous vegetation landscapes” model can be applied to synergistically enhance ecological functions and landscape value [5,23].

## 4. Materials and Methods

### 4.1. Study Area

**Figure 15:**
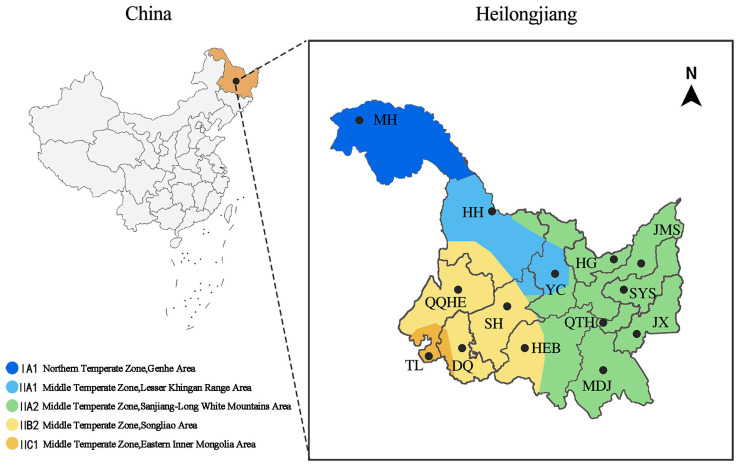
Study area and climatic subzone division.

Heilongjiang Province is located in the northeastern part of China, within the cold temperate zone (43°26′–53°33′ N, 121°11′–135°05′ E). It experiences a typical temperate continental monsoon climate, characterized by long, cold winters and short, humid summers, with significant climatic gradients across the region. Based on multi-year average temperature and aridity index, Heilongjiang Province is subdivided into five climatic subzones. To comprehensively cover urban habitat types under different climatic conditions, this study included 14 cities, encompassing all prefecture-level cities in Heilongjiang Province, as baseline sampling sites. This approach established a spatially extensive and climatically heterogeneous sample system of cold-region cities (Figure 15).

### 4.2. Survey Methods for Spontaneous Plants

**Figure 16:**
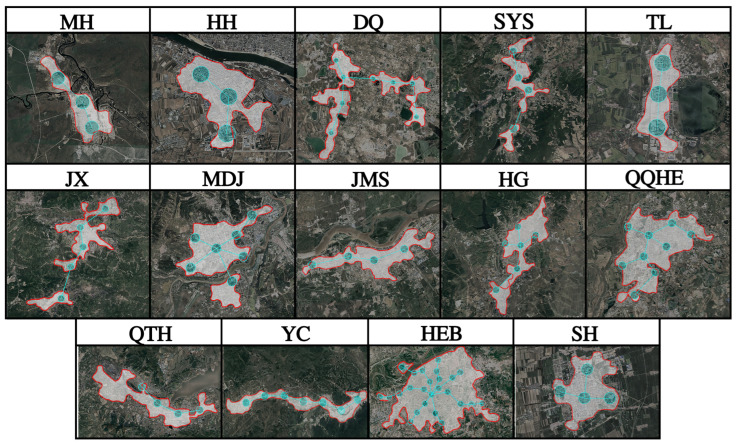
Sampling plots in 14 cities.

Based on the built-up area morphology of the 14 selected cities, survey transects were established. Along each transect, sampling plots with a radius of 500 m were set at approximately 3 km intervals. Within each plot, no fewer than six green space patches were selected for investigation (Figure 16). All field surveys were conducted during the peak growing season (June to September) across both 2023 and 2024 to comprehensively capture the spontaneous flora. We acknowledge that the number of plots per city varied due to differences in city size and morphology, which may introduce some limitations in cross-city comparisons. Standard quadrats were set according to vegetation type: 20 m × 20 m for the tree layer, 5 m × 5 m for the shrub layer, 1 m × 1 m for the herb layer. The Zurich-Montpellier school of phytosociological methodology was adopted to record species composition, coverage, height, and other relevant information. Habitat types were categorized into five types based on field characteristics and previous classifications, including lawn (LA), shrub-grassland gap (SG), forest gap (FG), soil-type abandoned land (SA), gravel-type abandoned land (GA) [26].

### 4.3. Data Processing

#### 4.3.1. Classification of Community Types

**Figure 17:**
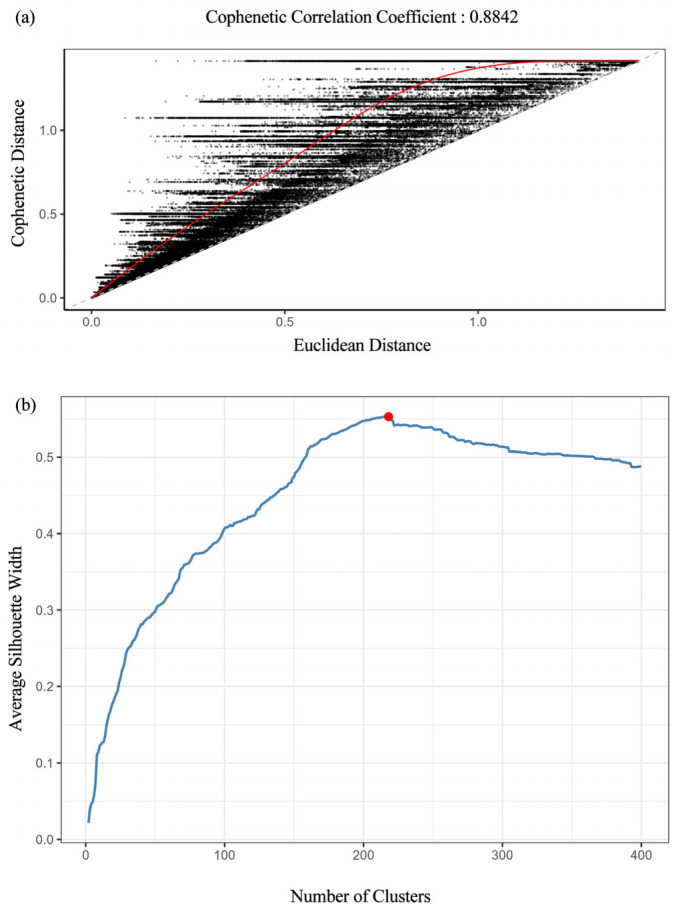
(**a**) Cophenetic correlation analysis; (**b**) Optimal number of clusters. Note: The red line shows the change in cophenetic correlation coefficient, and the red dot indicates the selected optimal cluster number at the “elbow” of the curve.

Relative dominance data of species in quadrats were standardized using R language and its “vegan” packages. A Euclidean distance matrix was calculated to quantify dissimilarities between quadrats. Hierarchical clustering was then performed using the complete linkage method. The cophenetic correlation coefficient was computed to evaluate the rationality of the clustering results. The optimal number of clusters was determined using the silhouette width method, based on which the quadrats were classified into distinct community types (Figure 17).

#### 4.3.2. Calculation Methods of Diversity Indices

(1) Species Dominance

The formula for calculating relative species dominance is as follows [60]:

(1) $$D=\left(IV/\sum IV\right)\times 100\%,$$

(2) IV=H×C,

where *D* is the relative dominance of the species in the community; *IV* is an important value; *H* is the maximum height of the species; *C* is the coverage of the species.

(2) *α*-diversity

Richness index is as follows [61]:

(3) R=S,

Shannon-Wiener diversity index is as follows [62]:

(4) $$H^{\prime }=-\sum_{i=1}^{S}p_{i}lnp_{i},$$

Pielou evenness index is as follows [63]:

(5) $$J=H^{\prime }/lnS,$$

where *S* is the number of species present in the community; *P_i_* is the relative dominance of species *i*.

(3) *β*-diversity

Jaccard index is as follows [64]:

(6) $$J=a/\left(a+b+c\right),$$

where *a* is the number of species present in both sampling plots; *b* is the number of species found only in the first sampling plot; *c* is the number of species found only in the second sampling plot.

Bray–Curtis index is as follows [65]:

(7) $$BC_{ij}=\frac{\sum_{k=1}^{S}\left|x_{ik}-x_{jk}\right|}{\sum_{k=1}^{S}\left(x_{ik}+x_{jk}\right)},$$

where *x_ik_* and *x_jk_* are the relative dominance of species *k* in sampling quadrat *i* and sampling quadrat *j*, respectively; *S* is the total number of species.

## 5. Conclusions

This study demonstrates that the diversity and composition of spontaneous plant communities in cold-temperate cities are governed by multi-scale drivers, where climatic zonation sets the macro-scale template, local habitat heterogeneity filters community assembly, and plant life forms reflect key adaptive strategies to environmental stress. These findings align with a multi-pathway ecological assembly framework.

The results offer critical implications for urban planning and biodiversity management. Firstly, the foundational role of the climatic gradient underscores that vegetation planning in northern cities like Helsinki or Toronto must prioritize regional hydrothermal conditions. Secondly, the significant influence of habitat heterogeneity suggests a shift from mere species introduction to the integrated management of the urban habitat mosaic. Consciously preserving or creating semi-natural habitats such as forest gaps and shrub-grassland complexes can effectively enhance habitat diversity for native biota and increase community stability. Furthermore, the adaptive strategies reflected by the life-form structure provide guidance for management practices; for instance, tailoring mowing frequency and soil disturbance levels can steer community succession toward more resilient trajectories. Finally, although invasive species constitute a limited portion of the total flora, their higher proportion within the non-native species pool in specific habitats calls for targeted monitoring and early intervention.

In summary, urban biodiversity conservation should be grounded in the holistic management of ecological processes and habitats. Future research should integrate metrics such as functional and phylogenetic diversity with long-term monitoring to deepen our understanding of spontaneous plant communities’ responses to global change, thereby providing a more robust scientific foundation for building sustainable and resilient urban ecosystems.

## Data Availability

The data presented in this study are openly available in Figshare at https://doi.org/10.6084/m9.figshare.30068152.

## Supplementary Materials

The following supporting information can be downloaded at: https://doi.org/10.6084/m9.figshare.30068197 (accessed on 6 September 2025), Figure S1: Split Dendrogram of Sample Clustering.

## Abbreviations

MH: Mohe; HH: Heihe; YC: Yichun; QQHE: Qiqihar; DQ: Daqing; SH: Suihua; HEB: Harbin; HG: Hegang; JMS: Jiamusi; SYS: Shuangyashan; QTH: Qitaihe; JX: Jixi; MDJ: Mudanjiang; TL: Tailai; HRB: Harbin; LA: lawn; SG: shrub-grassland gap; FG: forest gap; SA: soil-type abandoned land; GA: gravel-type abandoned land; IA1: Northern Temperate Zone, Genhe Area; IIA1: Middle Temperate Zone, Lesser Khingan Range Area; IIA2: Middle Temperate Zone, Sanjiang-Long White Mountains Area; IIB2: Middle Temperate Zone, Songliao Area; IIC1: Middle Temperate Zone, Eastern Inner Mongolia Area.
